# Revisiting the one in four: the prevalence of psychiatric disorder in the population of England 2000–2014

**DOI:** 10.1192/bjp.2019.196

**Published:** 2019-09-05

**Authors:** Paul E. Bebbington, Sally McManus

**Affiliations:** 1 Emeritus Professor of Social and Community Psychiatry, Division of Psychiatry, Faculty of Brain Sciences, University College London, UK; 2 Associate, Survey Research Centre, National Centre for Social Research, UK

**Keywords:** Mental disorders, epidemiology, prevalence, temporal change, national survey

## Abstract

Mental health problems are often said to affect one in four people in Britain, although with no consistent explanation of what the figure includes. We used three English national population surveys of psychiatric morbidity from 2000, 2007 and 2014 to provide prevalence rates for recent psychiatric problems. We combined disorders progressively to demonstrate the effects of cumulation. Psychosis had a prevalence of around 1%, severe common mental disorders added about 8%, and including less-severe common mental disorders gave a value around one in six. The figure of one in four required the inclusion of various other disorders. These values were strikingly stable over the surveys.

The prevalence one in four has been used extensively in service, policy and media contexts to quantify the extent of mental health problems in the British general population.^1^ Ginn and Horder^1^ attempted to identify the origins of this figure and point out a lack of any consistent determination of what the figure should include. It represents a collision between psychiatric epidemiology and the popular understanding of the meaning of mental disturbance: what categories would be acceptable examples of mental disorder to members of the populace? As Ginn and Horder^1^ suggest, the figure has social as well as scientific functions: demonstrating the relevance and significance of the phenomenon of mental illness, encouraging the funding of supportive services, reducing stigma by pointing out its considerable frequency and gaining acceptance of a plausible value. For this reason, any such figure should be examined in relation to its origins and assumptions: there may be conflict between what people know or believe, and what they are being told. There are thus cultural dangers both in overselling and in underselling the frequency of mental illness.

In our view, using an undefined concept risks loss of credibility and the meaning of this figure should therefore be spelt out. The British Adult Psychiatric Morbidity Survey (APMS) programme has applied standardised and structured methods for identifying individual mental disorders in regularly repeated surveys based on randomly chosen samples of the household population. We used the surveys carried out in 2000, 2007 and 2014^2–4^ to log both an overall burden of disorder (i.e. the proportion of people with one or more mental health problems) and changes in its magnitude.

## Method

There are difficulties in presenting information about overall prevalence. The first concerns the disorders to be covered. There is little dispute about the inclusion of common mental conditions such as anxiety and depressive disorder, and rarer but severe disorders like psychosis and bipolar disorder. Other disorders recognised in international classifications are also candidates for inclusion, for instance post-traumatic stress disorder (PTSD) and attention-deficit hyperactivity disorder (ADHD). Likewise, including dependence on illicit drugs and alcohol is unlikely to be contentious. However, in the analyses presented here, we excluded the hazardous use of alcohol and nicotine from analysis because, although clearly a behavioural problem and a social concern, it may not conform to lay ideas of mental disorder. The inclusion of hazardous use would of course result in higher values for the prevalence of mental disorder, to a degree that might be treated with suspicion by potential consumers of this information.

The new analyses presented here are based on the three most recent APM surveys, carried out in 2000, 2007 and 2014.^2–4^ The two later surveys cover only England, and we therefore restrict the analyses of the 2000 survey to respondents from England. Structured interviews were used to provide a logical, valid and clinically meaningful classification, thereby formalising the process of clinical description and case identification. These measures were not adjusted to accommodate intervening changes in the dominant classificatory schemes^5,6^ and are thus comparable across surveys. They are referenced and described in detail in the reports.^2–4^ The values presented here are based on point prevalence for common mental disorders (CMDs) and a prevalence period of up to 1 year for the other disorders. Again, this follows the procedures adopted in the reports.

Comorbidity between psychiatric disorders is common, with the consequence that adding in a new category increases the overall percentage of people affected by less than the individual prevalence of that category. For this reason, we quantified people suffering from mental illness by incorporating disorders sequentially. Such a sequence must inevitably have an arbitrary element. We chose to start with psychotic disorder and CMDs, then moving on to include dependence disorders, as these were quantified identically in all three surveys. Disorders assessed only in the later surveys were included later in the sequence of analysis.

We first identified those suffering from probable psychosis. From the remaining group, we then added people with an identified CMD, in sequence according to their severity. Severity was estimated from their score on the instrument used to identify them, the revised Clinical Interview Schedule.^7^ Scores of 12–17 indicate a significant mental health problem requiring assessment, whereas scores of ≥18 imply treatment is almost certainly needed. From the remaining sample, we then incorporated people with signs of alcohol or drug dependence.^8,9^ Next, we added those who screened positive for ADHD,^10^ and finally people screening positive for personality disorder,^11^ PTSD^12^ or bipolar disorder.^13^ The ADHD screen was not used until the 2007 survey, and the personality disorder, PTSD and bipolar disorder screens were only introduced in 2014.

## Results

The results are set out in detail in Table 1. The most severe disorder, psychosis, had a prevalence of around half of 1% in the two earlier surveys, but this rose significantly to exceed 1% in 2014. (As the screen definition for psychosis includes being on antipsychotic medication, increased prescribing might have been responsible for this apparent increase in prevalence.) However, the values based on the inclusion of the other types of mental disorder are remarkable for their stability. This is driven to a considerable extent by the virtually unchanging overall frequency of CMDs. Depending on the severity threshold used to include CMDs, combining them with psychosis yields a value of one in ten or one in six. The addition of substance use disorders raises this to just over one in five, and again the values are constant over time. A value of one in four is achieved by adding in the results of screening for ADHD. Finally, incorporating personality and other disorders boosts the figure to around one in three.

**Table 1:** The prevalence of mental disorders in England 2000–2014

Category	Disorders	Year					
		2000		2007		2014	
		%	95% CI	%	95% CI	%	95% CI
Severe mental illness	Psychosis in past year	0.6	0.4–0.8	0.5	0.3–0.7	1.1^a^	0.9–1.4
Plus severe CMD cases	CIS-R score ≥18 in relation to past week	8.1	7.4–8.8	8.1	7.4–8.8	9.0	8.2–9.9
Plus all CMD ‘cases’	Current CMDs (CIS-R score ≥12 in relation to past week)	17.6	16.6–18.7	17.0	16.0–18.1	18.2	17.1–19.3
Plus substance use disorders	Harmful/dependent pattern of alcohol or signs of drug dependence in past year	22.3	21.1–23.4	21.0	19.9–22.2	22.0	20.8–23.2
Plus developmental disorders	ADHD-positive screen	NA		24.5	23.3–25.8	26.1	24.8–27.4
Plus personality and other disorders	SAPAS (personality disorder screen), PTSD screen, bipolar disorder screen	NA		NA		31.6	30.2–32.9

CMD, common mental disorder; CIS-R, Clinical Interview Schedule – Revised; ADHD, attention-deficit hyperactivity disorder; SAPAS, Standardised Assessment of Personality – Abbreviated Scale; PTSD, post-traumatic stress disorder; NA, not available.

Cumulative totals presented. Based on 16- to 74-year-olds living in England, data weighted to represent the national population.^2–4^ See McManus *et al*^4^ for details of measures.

a. As the screen definition for psychosis includes being on antipsychotic medication, increased prescribing may have been responsible for some of this increase in prevalence.

## Discussion

We found that psychosis had a prevalence of around 1%, severe CMDs added about 8% and including less-severe CMDs gave a value around one in six. The figure of one in four required the inclusion of various other disorders.

A major purpose of the APMS programme is to provide information on temporal changes in the prevalence of a range of different disorders, with the expectation that these may be related to a changing social context. Problems in identifying disorders in the general population also arise from the fact that symptoms of mental illness are exponentially distributed. Imposing categories on continuous distributions is always a fraught process. So, for example, although many people have a few isolated affective symptoms, relatively few have sufficient symptoms to justify a diagnosis of mental disorder.^14^ This problem also applies to paranoia^15^ and personality disorder.^16^

There is an inevitable arbitrariness both in selecting the mental conditions for consideration, and in selecting the order in which disorders are entered. The number of disorders determines the number of people who will be identified as suffering from some kind of disorder, whereas the order of entry determines the values of intermediate combinations of disorder. Our choices were based on judgements about how clinicians would probably target treatment in circumstances of comorbidity.

The most striking finding from these analyses is the absence of secular change: for people aged 16–74 years, the prevalence of mental disorders in England over a prolonged period remained remarkably constant. What does this imply? The most parsimonious inference is that the determinants of mental disorder, social or biological, have themselves not varied over that period. However, this would be surprising, certainly given the very considerable changes in social conditions and social behaviour in England since the millennium. A less parsimonious explanation is that there have been changes in a variety of influences bearing on the various disorder types, but that these have tended to cancel each other out. Such hypotheses are open to empirical testing, thereby underlining the importance of programmes like APMS for studying the nature of disorder and the implications for treatment and social policy. For example, changes between the 2014 survey and that planned for 2021 may make it possible to identify the mental health changes occurring in the context of intervening welfare policies characterised by austerity.
